# Combined analysis of proteomics and metabolism reveals critical roles of oxidoreductase activity in mushrooms stimulated by wolfberry and sea buckthorn substrates

**DOI:** 10.3389/fnut.2025.1543240

**Published:** 2025-03-18

**Authors:** Yuan Zhao, Hongying Li, Youhua Yao, Qing Wei, Tilong Hu, Xia Li, Boxu Zhu, Hailing Ma

**Affiliations:** ^1^College of Ecol-Environmental Engineering, Qinghai University, Xining, China; ^2^Academy of Agriculture and Forestry, Qinghai University, Xining, China; ^3^Laboratory for Research and Utilization of Qinghai Tibet Plateau Germplasm Resources, Xining, China

**Keywords:** proteomics, metabolism, wolfberry, sea buckthorn, *Lentinula edodes*, oxidoreductase activity

## Abstract

**Background:**

Cultivating edible fungi, particularly *Lentinula edodes*, efficiently transforms agroforestry byproducts into valuable products. However, the mechanism of the promotive effects of those substrates was largely unknown. This study used wolfberry (WB) and sea buckthorn (SBK) substrates to investigate mushroom fruiting bodies’ physiological, proteomics, and metabolism profiling.

**Results:**

Results show that compared to apple wood (AW), the crude protein and fatty acids were substantially enhanced by both WB and SBK treatment. We identified 1409 and 1190 upregulated and downregulated differentially abundant proteins (DAPs) for the SBK versus AW group and observed 929 overlapped DAPs with upregulation patterns. Of these DAPs, carbohydrates and oxidoreductase activity pathways were significantly enriched. Moreover, the enhanced expression of nine genes by WB and SBK was confirmed by qPCR. Metabolism suggests that 66 differentially abundant metabolites overlapped in the list of two comparison groups (WB versus AW and SBK versus AW).

**Conclusion:**

Collectively, we summarized that both WB and SBK stimulate glucose degradation, enhance the expression of gene-related oxidoreductase activity, and promote protein biosynthesis by coordinating with amino acid metabolism. This study highlights the importance of oxidoreductase activity in promoting nutritional value in mushroom fruiting bodies induced by WB and SBK substrates.

## Introduction

Cultivating edible fungi is an efficient biological method for transforming agroforestry byproducts into valuable mushroom products, directly linking lignocellulosic waste to human food consumption (1, 2). Various substrates are extensively used in mushroom cultivation. For instance, using waste apple wood as sawdust for cultivating *Pleurotus ostreatus* and *Lentinula edodes* is common (3). Furthermore, *Korshinsk peashrub* (*Caragana korshinskii* Kom.) is utilized as a substrate element for cultivating various *Pleurotus* spp., including *Pleurotus eryngii*, *Pleurotus ostreatus* and *Pleurotus tuoliensis* (4–6). Another notable example is sea buckthorn (*Hippophae rhamnoides* L.), a deciduous shrub rich in nutrients such as oleic acid, protein, amino acids, and potassium. It is valuable for nutritional and therapeutic purposes (7, 8). *Lycium barbarum* L. (wolfberry), a well-known Chinese herb, offers numerous nutritional and functional benefits due to its diverse components. These components include amino acids, polyphenols, carotenoids, polysaccharides, organic acids, fatty acids, phytosterols, and their derivatives (9, 10).

However, studies on using sea buckthorn (SBK) and wolfberry (WB) as substrates for mushroom cultivation are limited, primarily due to local practices and production needs. The introduction of these novel substrates for *Pleurotus* spp. cultivation encourages the effective utilization of lignocellulosic waste and helps mitigate the scarcity of conventional raw materials, such as cottonseed hulls. Our preliminary research indicates that sea buckthorn and wolfberry offer several advantages for mushroom cultivation, making them promising candidates for substrate resources. Despite their potential, developing mushroom fruiting bodies is a highly complex process. Various cellular mechanisms, genetic factors, physiological aspects, and environmental influences regulate this process, all vital in ensuring successful fruiting (11). For example, cellular redox homeostasis is an essential and dynamic process that ensures the balance between reducing and oxidizing reactions within cells, which comprises an extensive network of oxidoreductase enzymes, small antioxidants, and reactive species regulating mushroom development (12). However, the molecular mechanisms by which these two substrates influence mushroom development remain poorly understood.

In recent years, mass spectrometry (MS)-based omics approaches, including proteomics and metabolism analysis, have become widely used to study global protein and metabolite profiles. These approaches have revealed key biosynthetic pathways that control plants’ temporal growth and development (13, 14). Despite this, relatively few studies address the metabolic differences among mushrooms across different spatial dimensions and cultivars (15, 16). Consequently, integrating metabolomics and proteomics is a powerful method for understanding the complex metabolism and molecular mechanisms of gene regulation by constructing a unified pathway (17–19). This integrated approach has been extensively applied to various microbes and higher plants, such as Arabidopsis, tomato, and alfalfa, to uncover biosynthesis mechanisms of functional bio-actives, including flavonoids, polyketides, and terpenoids (20, 21). Integrative analyses have been particularly effective in investigating the relationship between microbial activity and metabolite degradation during anaerobic digestion (17–19). However, most omics analyses of *L. edodes* have concentrated on the developmental process from mycelium to fruiting bodies (22, 23), with limited reports on integrated transcriptomics and metabolomics analyses of malformed *L. edodes* fruiting bodies.

In this study, we conducted a comprehensive analysis by integrating proteomics and metabolomics assessments of mushroom fruiting bodies cultivated on SBK and WB substrates and compared them to apple wood sawdust (AW) substrates. This study aimed to furnish preliminary data, deciphering key metabolic pathways and genes responsive to the distinct effects of substrates in mushrooms. Additionally, qPCR was employed to validate changes in key genes within the mushroom samples with SBK and WB substrates against AW. Identifying key regulatory factors for mushrooms with the two substrates will help guide precise gene editing strategies, ultimately enhancing the economic benefits of mushroom production.

## Materials and methods

### Materials and growth conditions

This study used the mushroom variety L808, which is widespread in Northwest China. The experiment was conducted at a shiitake mushroom production base in Menyuan County, Qinghai Province (37°39′N, 101°63′E, altitude 3,700 m) from January to December 2019. The region exhibits a continental plateau climate with a mean temperature of 1.3°C and yearly precipitation of 530-560 mm.

We used three types of substrates for fruit trees: SBK, WB, and AW. Therefore, two cultivation schemes were constructed (SBK group: 78% SBK, 20% bran, 1% gypsum powder, 1% sucrose; WB group: 78% WB, 20% bran, 1% gypsum powder, 1% sucrose; AW group: 78% apple wood, 20% bran, 1% gypsum powder, 1% sucrose). Standard management methods for fruiting were used (24). In each group of woody substrates, 10 fruiting bodies were identified based on relatively uniform maturation, absence of mechanical damage, cap closure (no open umbrellas), absence of disease signs, and random selection of uniform size. The basal medium was removed, cut into 3 mm thick pieces, and mixed in one replicate for 6 replicates, resulting in 12 samples and rapidly frozen in liquid nitrogen.

### Measuring nutritional properties

Free fatty acids, crude protein, and total fiber content were measured using the Society of Analytical Chemistry method (25) with slight modifications (26). In summary, dyes, tiny sugar molecules, and contaminants were eliminated from 2 g of powdered fruiting bodies by extracting them with 80 mL of 80% ethanol at 90°C for 1 h. Then, the insoluble residue was dried and extracted twice more for 1 h each time, using 80 mL of distilled water at 90°C. The extract was filtered and centrifuged at 4,500 × *g* for 15 min to obtain a filtrate.

### Sample preparation through protein extraction and filtration

Mushroom fruiting bodies were collected after cultivating on SBK, WB, and AW substrates for 21 days. The samples underwent instant freezing in liquid nitrogen and then were crushed using a pestle and mortar. After precipitating with TCA/acetone buffer, SDT buffer (4% SDS, 100 mM DTT, 150 mM Tris–HCl pH 8.0) containing protease inhibitors were added. Protein concentration was measured using a BCA protein assay kit (Pierce, Thermo, USA). Using a Microcon (10 kDa), samples were re-ultrafiltered with UA buffer (8 M Urea, 150 mM Tris–HCl pH 8.0) to exclude surfactants and other low molecular weight substances. Subsequently, 30 min were spent in the dark with the samples after adding 100 μL iodoacetamide (100 mM IAA in UA buffer) to inhibit reduced cysteine residues. After that, the filters were cleaned twice with 100 μL 25 mM NH_4_HCO_3_ buffer and three times with 100 μL of UA buffer. Using 4 μg trypsin (Promega) in 40 μL 25 mM NH_4_HCO_3_ buffer, proteins were digested overnight at 37°C. The resultant peptides were then collected as a filtrate. Using C18 filter Cartridges (Empore SPE C18 cartridges, standard density, 7 mm inner layer diameter, 3 mL volume, Sigma), the peptides from each sample were desalted. They were then concentrated using vacuum centrifugation with 40 μL of 0.1% (v/v) formic acid. It was dissolved and reorganized with moderate reconstruction. Tryptophan and tyrosine residue in vertebrate proteins were used to determine the peptide content. UV spectral density at 280 nm was measured with the extinction coefficient 1.1 for 0.1% (g/L) solution.

### Mass spectrometry with data-independent acquisition (DIA)

Analyzing each sample’s peptides, Shanghai Applied Protein Technology Co., Ltd. used LC–MS/MS in data-independent acquisition (DIA) mode. Each DIA run comprised one complete MS–SIM scan and 30 DIA scans spanning the mass range of 350–1800 m/z. The configurations are as follows: Complete SIM card scan in contour mode with resolutions of 120,000 and 200 m/z, AGC set to 3 × e^6^, and a maximum IT times of 50 ms. The resolution of the DIA scans is 15,000, the AGC target is 3 × e^6^, the maximum IT is automatically set, and the normalized collision energy is 30 eV. Using a linear gradient of buffer B (80% acetonitrile and 0.1% Formic acid) at a flow rate of 250 nL/min, the experiment took 90 min in total. A pooled aliquot of each experimental sample was injected for 6 injections at the beginning of the MS study and within DIA mode to track the functioning of the MS system. These samples served as quality control (QC) samples. Using the created spectral library, DIA data were examined in Spectronaut (v2.13) and contrasted with the *Lentinula edodes* protein database (https://www.uniprot.org/taxonomy/4751). Important software functions include cross-run normalization, MS2-level interference correction capability, and dynamic iRT for retention time prediction. The following selection thresholds were applied to collect positive hits: mass accuracy, 5 ppm; Dcn, 0.1; primary score, 200; rsp., 5; those peptides having scores (XC) above 1.5, 2.0, and 2.5 for ion charges at +1, +2, and + 3 and higher were automatically selected as positives for identification. The threshold of the Mascot is set to be 20%. A Q-value cutoff of 0.01 was used to filter the results, meaning an FDR < 1% was achieved.

### Enrichment of GO functions and KEGG pathway

Based on differentially expressed proteins (DAPs) revealed in mushroom fruiting bodies cultivated on SBK and WB substrates, Gene Ontology (GO) functional enrichment and Kyoto Encyclopedia of Genes and Genomes (KEGG) pathway enrichment were carried out and identified for AWS analysis as comparison targets. Protein sequences of the DAPs were searched locally using NCBI BLAST+ client software and InterProScan to identify homologous sequences. GO terms were mapped, and the arrays were annotated using Blast2GO software. Visualize the GO annotation results using an R script. These proteins were then screened against the Kyoto Encyclopedia of Genes and Genomes (KEGG) database (http://geneontology.org/) to identify KEGG orthologs and map them to KEGG pathways.

### Verification of DEGs by qPCR

qPCR experiments were performed to validate the gene expression results obtained from the proteomics analysis. Total RNA was extracted using Trizol Reagent (Thermo Fisher, Carlsbad, CA, USA) according to the manufacturer’s instructions. The RNA concentration was measured using a NanoDrop spectrophotometer. qRT-PCR confirmed eight common DAPs in mushroom fruiting bodies cultured in WB and SBK. Details of the primers are given in Supplementary Table S1. PrimeScript RT Master Mix (Perfect Real Time, Takara, Shanghai, China) and the miRNA RT-PCR equipment (BioTNT, A2030A001-120 T, Hangzhou, China) were used to reverse transcribe the extracted RNA into cDNA, resulting cDNA was stored at −20°C (27). The qPCR reaction mixture consisted of 10 μL UNICONTM qPCR SYBR Green Master Mix, 1 μL primer mix (200 nM), 6 μL cDNA template and 6 μL of RNase-free ddH2O. The thermal cycle program was set up as follows: 40 cycles were carried out at 95°C for 3 min, 95°C for 10 s, 65°C for 20 s, 72°C for 30 s. the final stage consisted of 1 min at 65°C and 15 s at 95°C. Relative mRNA expression levels were calculated using the 2^−ΔΔ*Ct*^ method, comparing 16S rRNA as the reference gene. To ensure the reproducibility and reliability of the results, every reaction was carried out three times.

### Metabolism determinations

Untargeted metabolic analysis of mushroom fruiting bodies cultured on SBK and WB substrates using AWS was performed using LC–MS/MS and a Triple Quad 6,500 SCIEX system (28). After gathering around 2.5 mg of mushroom fruiting bodies into 2 mL Eppendorf tubes with metal beads chilled beforehand, the tubes were instantly frozen in liquid nitrogen. First, the samples were ball milled for 5 min at 30 Hz. After dissolving the resultant powder in 1.5 mL of methanol/chloroform, it was incubated for 5 h at −20°C. Following incubation, the mixture was filtered using an organic phase filter (GE Healthcare, 6,789–0404) with 0.43 μm size and centrifuged for 10 min at 2000 g and 4°C.

Metabolomic analysis was carried out using Metabolomic software (Durham, NC, USA). Retention periods and mass spectra were compared with reference metabolites to identify the components of the sample. To precisely identify metabolizable substances in each sample, mass spectra using NIST02 records and then the Golm Metabolome Database (http://csbdb.mpimp-golm.mpg.de/csbdb/gmd/gmd.html) were utilized. The student *t*-test found differential abundant metabolites (DAMs) based on peak signal intensity between each comparison group (SBK vs. AW or WB vs. AW).

## Results

**Figure 1:**
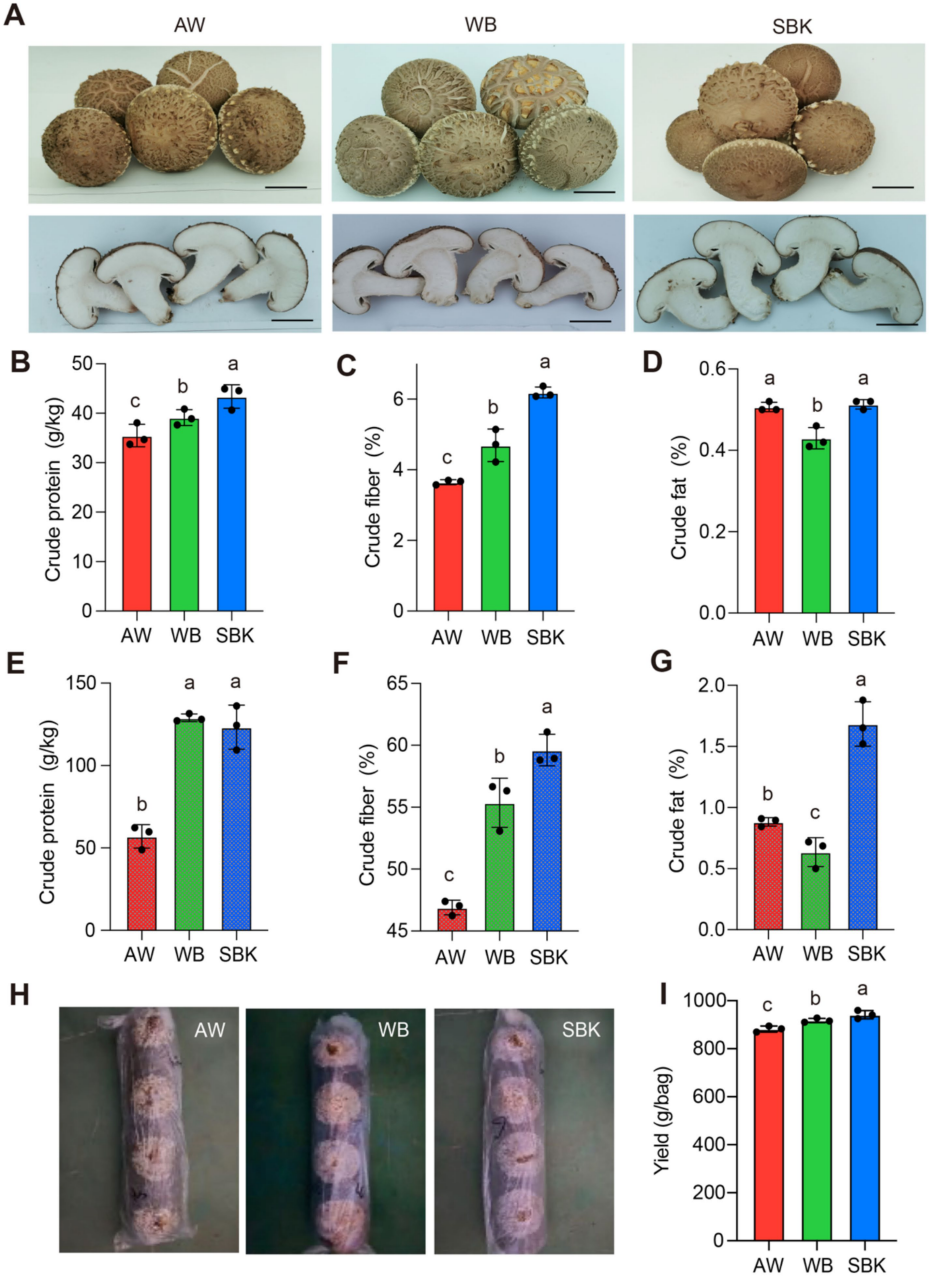
Physiological features of mushroom fruiting bodies cultivated in apple wood, wolfberry, and sea buckthorn. **(A)**, images of mushroom fruiting bodies cultivated with different substrates. The scale of the bar is proportional to the length of the mushroom bodies. AW, apple wood, WB, wolfberry; SBK, sea buckthorn. **(B–D)**, crude protein, crude fiber, and crude fat determined in mushroom fruiting bodies induced by different substrates (WB, SBK, and AW). **(E–G)**, crude protein, crude fiber, and crude fat in three substrates (WB, SBK, and AW). *n* = 3. **(H),** images of representative mushroom phenotypes cultivated with three substrates. **(I),** yield per bag in mushroom bodies cultivated with three substrates. Based on one-way *ANOVA*, different letters represent the significant differences for each trait among the three substrates.

In this study, we analyzed the promotive effect of two wood sawdust substrates (SBK and WB) compared to AW on the development of the mushroom (*Lentinula edodes*). The fruiting body sizes were not significantly altered among SBK, WB, and AW (Figure 1A). The crude protein content was enhanced by 10 and 22% for WB and SBK, respectively, relative to AW (Figure 1B), while crude fiber contents were stimulated by 28 and 70%, respectively (Figure 1C). However, crude fat contents showed similar or slightly decreased in mushroom fruiting bodies induced by WB and SBK compared to AW (Figure 1D). Regarding the contents of three physiological features in substrates, we observed the pattern was very similar to that in mushroom fruiting bodies (Figures 1E–G). The weight of mushroom bodies cultivated with WB and SBK was significantly increased than that with AW (Figures 1H,I).

We performed an integrative analysis of proteomics and metabolites to decipher the key molecular profiling changes in the mushroom fruiting bodies induced by different substrates. Based on proteomics, we observed that identified protein abundance across 24 samples from different substrates was all around 200,000 (Supplementary Figure S1), and principal component analysis revealed that PC1, PC2, and PC3 accounted for 45.7, 20.3, and 11.4%, which could clearly distinguish the samples from different groups (Figure 1A). These results suggest the effectiveness of proteomics for the current study. Subcellular localization analysis showed that for these DAPs in the comparison of WB vs. AW, there were 503 and 529 DAPs, with the largest proportion localized in the nucleus and cytoplasm (Supplementary Figure S2A). Similarly, there were 440 and 414 DAPs to compare SBK vs. AW, with the largest proportion localized in the nucleus and cytoplasm (Supplementary Figure S2B). Domain analysis revealed that most DAPs were annotated to contain a protein kinase domain and DEAD/DEAH box helicase compared to WB vs. AW (Supplementary Figure S3A). In contrast, most DAPs contained a protein kinase domain and helicase conserved C-terminal domain compared to SBK vs. AW (Supplementary Figure S3B).

**Figure 2:**
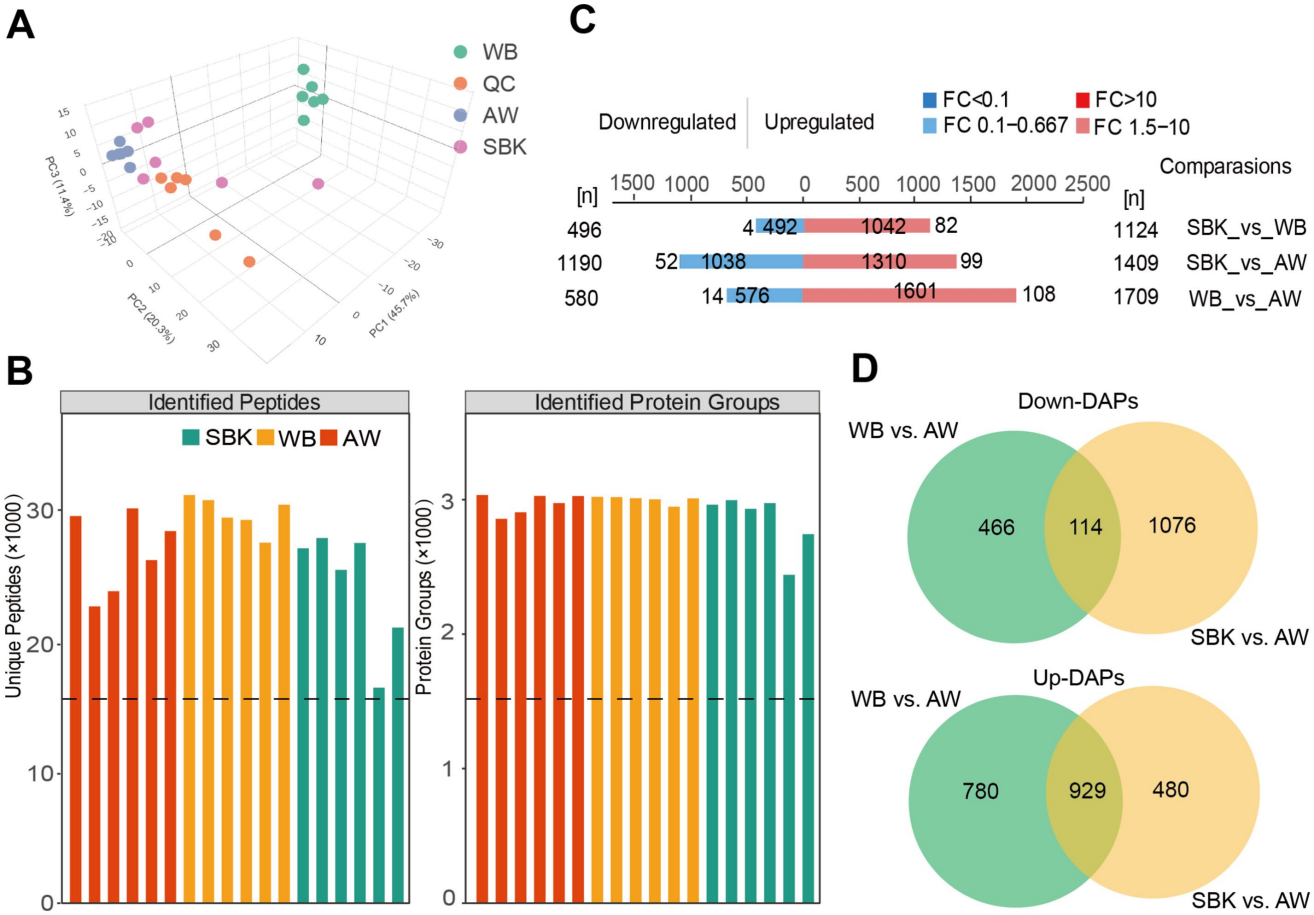
Proteomics analysis of mushroom fruiting bodies treated with SBK and WB relative to AW. **(A)**, principal component analysis on the 24 samples used in this study. QC: quality control samples. **(B)**, statistical analysis of the peptides and corresponding proteins in different groups. **(C)**, differentially abundant proteins in different comparisons based on proteomics analysis. **(D)**, Venn diagram showing the upregulated and downregulated DAPs in the comparison of either SBK or WB relative to AW.

Based on the proteomics analysis from 24 mushroom fruiting body samples, we identified up to 30,000 peptides and correspondingly identified 3,000 proteins (Figure 2B). We observed 1,124, 1,409, and 1709 upregulated DAPs for comparing WB vs. SBK, SBK vs. AW, and WB vs. AW, respectively (Figure 2B). In contrast, there were 496, 1,190, and 580 downregulated DAPs for the three comparisons (WB vs. SBK, SBK vs. AW, and WB vs. AW), respectively (Figure 2C). To uncover the key DAPs induced by the two substrates, we identified 114 downregulated DAPs that overlapped between the comparisons of WB vs. AW and SBK vs. AW (Figure 2D; Supplementary Table S2), and 929 upregulated DAPs that overlapped in the comparison of SBK vs. AW (Figure 2D; Supplementary Table S3).

**Figure 3:**
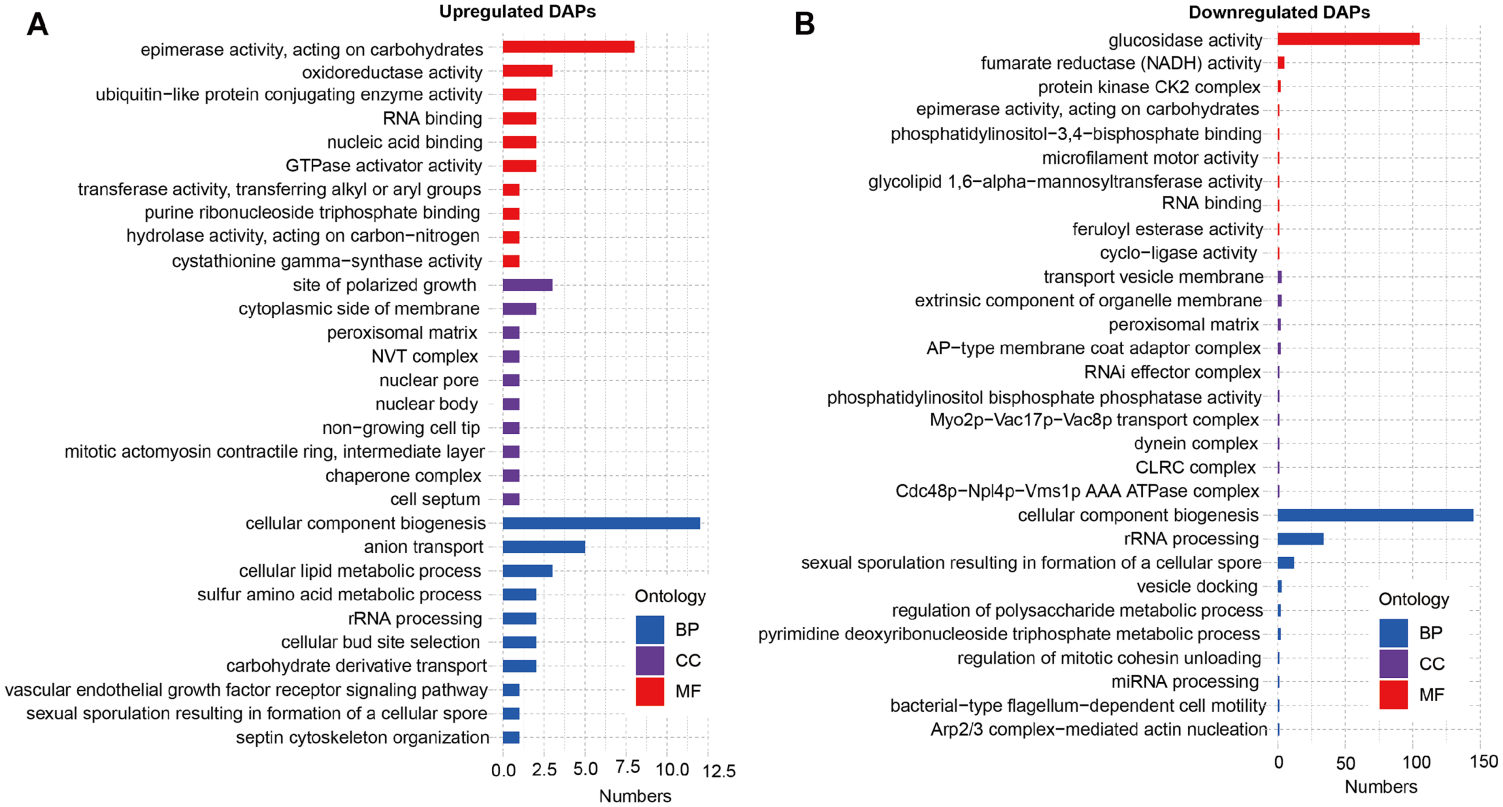
GO analysis on the list of overlapped differentially abundant proteins in the mushroom fruiting bodies induced WB or SBK relative to AW. **(A)**, downregulated DAPs. **(B)**, upregulated DAPs.

**Figure 4:**
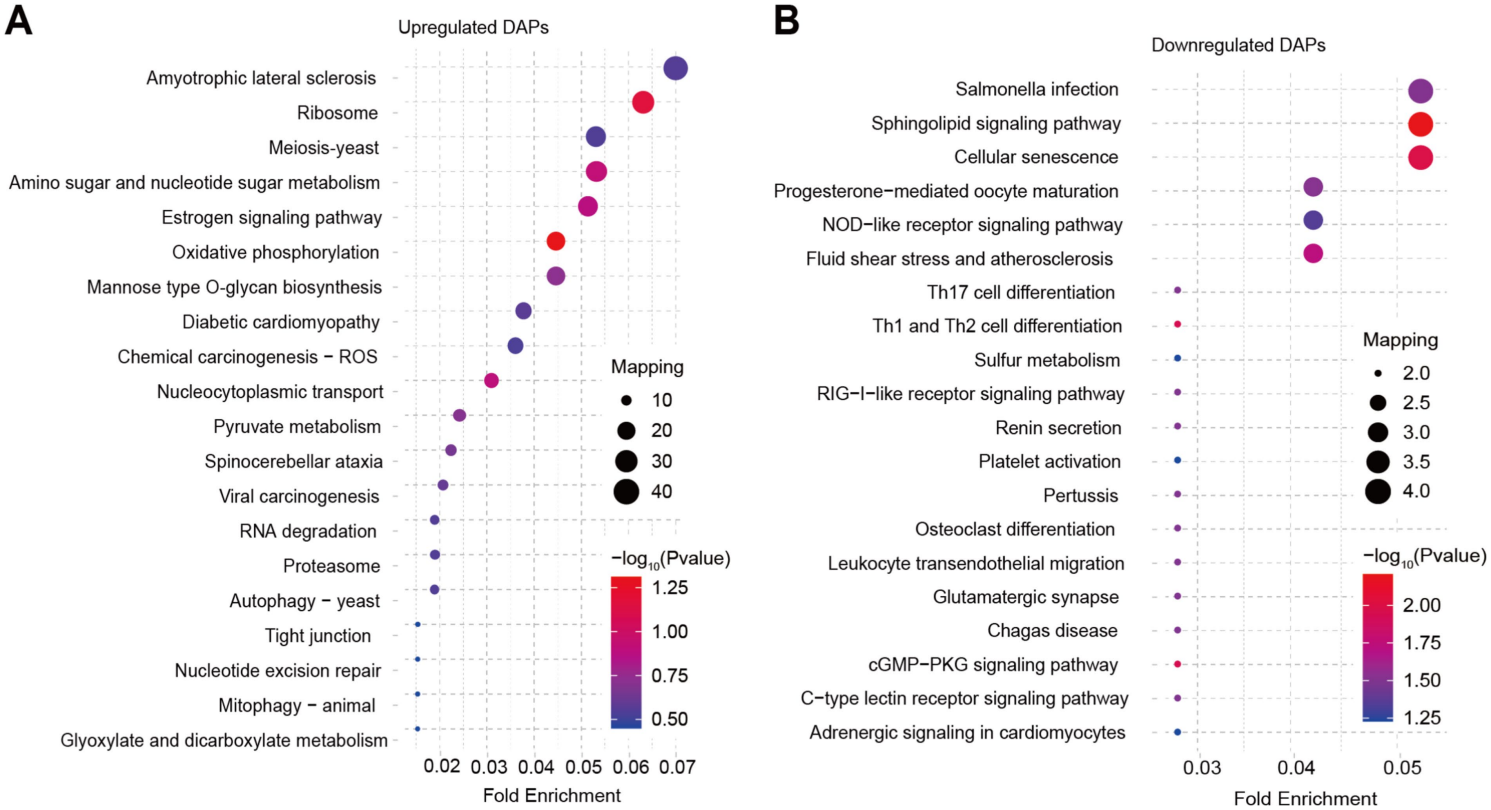
KEGG analysis on the list of overlapped differentially abundant proteins in the mushroom fruiting bodies induced WB or SBK relative to AW. **(A)**, upregulated DAPs. **(B)**, downregulated DAPs.

Furthermore, we performed GO and KEGG analysis on the list of 929 upregulated DAPs and 114 downregulated DAPs that overlapped between SBK vs. AW and WB vs. AW. GO analysis revealed that the list of upregulated DAPs significantly enriched epimerase activity, acting on carbohydrates, oxidoreductase activity, and cellular component biogenesis (Figure 3A). In contrast, glucosidase activity, fumarate reductase (NADH) activity, and protein kinase CK2 complex were significantly enriched in the list of downregulated DAPs (Figure 3B). Results based on KEGG analysis suggested that some pathways, such as oxidative phosphorylation and pyruvate metabolism, were significantly enriched in the list of upregulated DAPs for both comparisons (WB vs. AW and SBK vs. AW) (Figure 4A). In contrast, in the downregulated DAPs list, we found that some pathways, such as cellular senescence, NOD-like receptor signaling pathway, and Th17 cell differentiation, were significantly enriched (Figure 4B).

**Figure 5:**
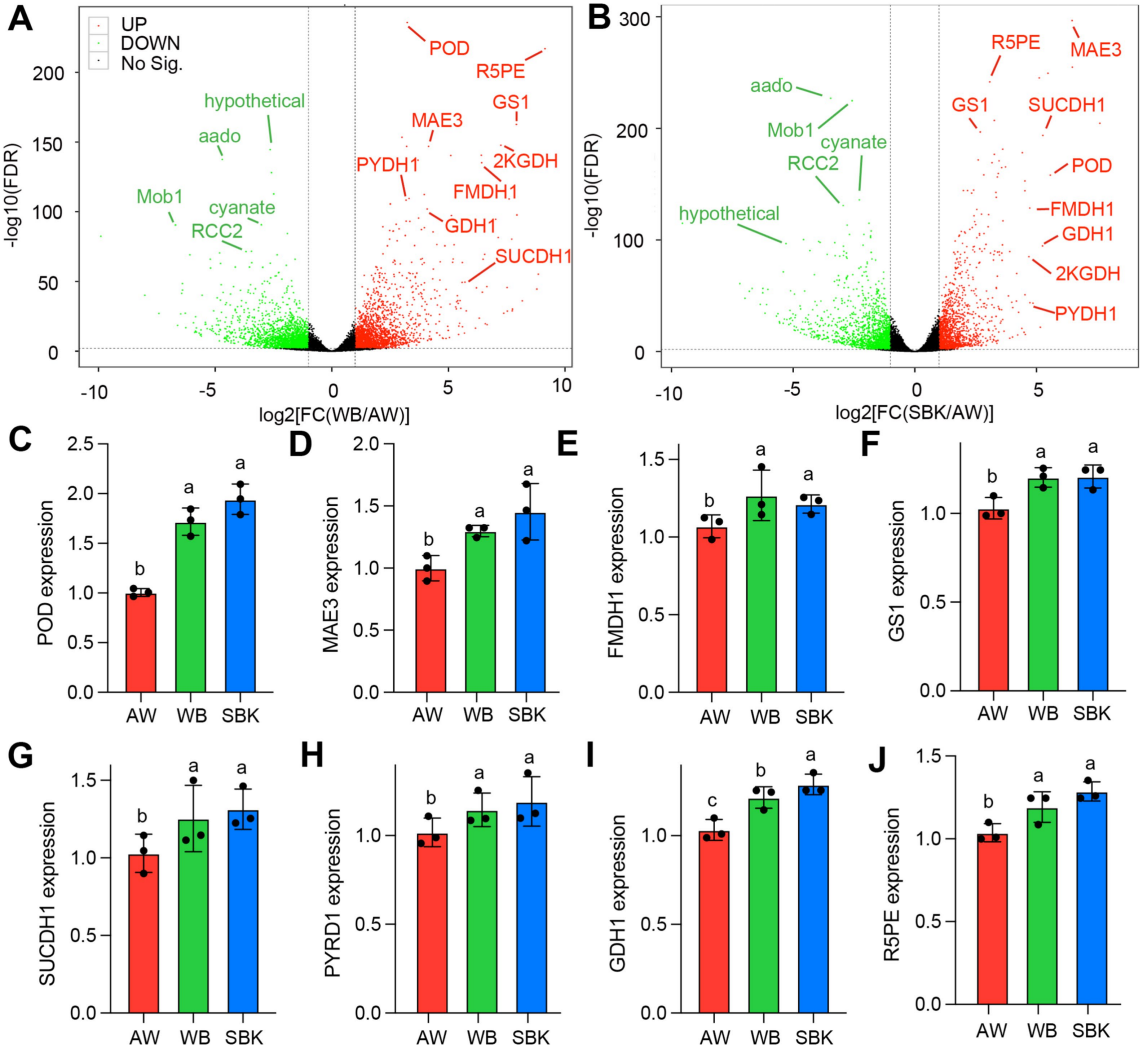
Overlapped differentially abundant proteins induced between WB and SBK against AW in mushroom fruiting bodies. **(A,B)**, volcano plot of the list of DAPs in either WB or SBK against AW. The overlapped differentially abundant proteins (DAPs) with extremely significant differences were highlighted in fonts with different colors. **(C–J)**, qPCR validation for the overlapped DAPs with upregulation patterns in WB or SBK relative to AW. Different letters represent the significant differences among WB, SBK, and AW in different mushroom fruiting bodies. Each vertical bar represents the mean *n* = 3 (±SE).

In particular, we identified that hypothetical, aado, Mob1, cyanate, and RCC2 were highly downregulated in the overlapped DAPs list (Figures 5A,B). POD, R5PE, GS1, MAE3, 2KGDH, PYDH1, FMDH1, GDH1, and SUCCDH1 were highly upregulated in the overlapped DAPs list between the two comparisons (SBK vs. AW and WB vs. AW) (Figures 5A,B). We further validated eight upregulated DAPs, including POD, R5PE, GS1, MAE3, PYDH1, FMDH1, GDH1, and SUCCDH1 (Supplementary Table S4). As expected, qPCR results supported the protein levels among the three substrate treatments (Figures 5C,J). Results based on proteomics suggest that the fold change in SBK vs. AW ranged from 1.3 to 20.3, while it ranged from 1.1 to 2.2 when comparing WB to AW (Supplementary Table S3).” In particular, the levels of POD (GAW09174.1) show 20.3 and 2 increases in fold change compared to SBK vs. AW and WB vs. AW, respectively (Supplementary Table S3). Regarding gene expression, the levels of the POD gene (LENED_011308) were increased up to 2 times fold change, exhibiting the highest enhancement among the eight genes detected by qPCR.

We performed a non-targeted metabolism analysis to illustrate further the changes in amino acids induced by the three substrates. Quality control based on non-targeted metabolism revealed the number of peaks with relative standard deviation (RSD) ≤30% in the QC sample accounted for more than 80% of the total number of peaks in the QC sample (Supplementary Figure S4A), indicating that the instrument analysis system was relatively stable. The data could be used for subsequent analysis. The identified metabolites mainly comprised organic acids, derivatives, and lipid-like molecules (Supplementary Figure S4B).

**Figure 6:**
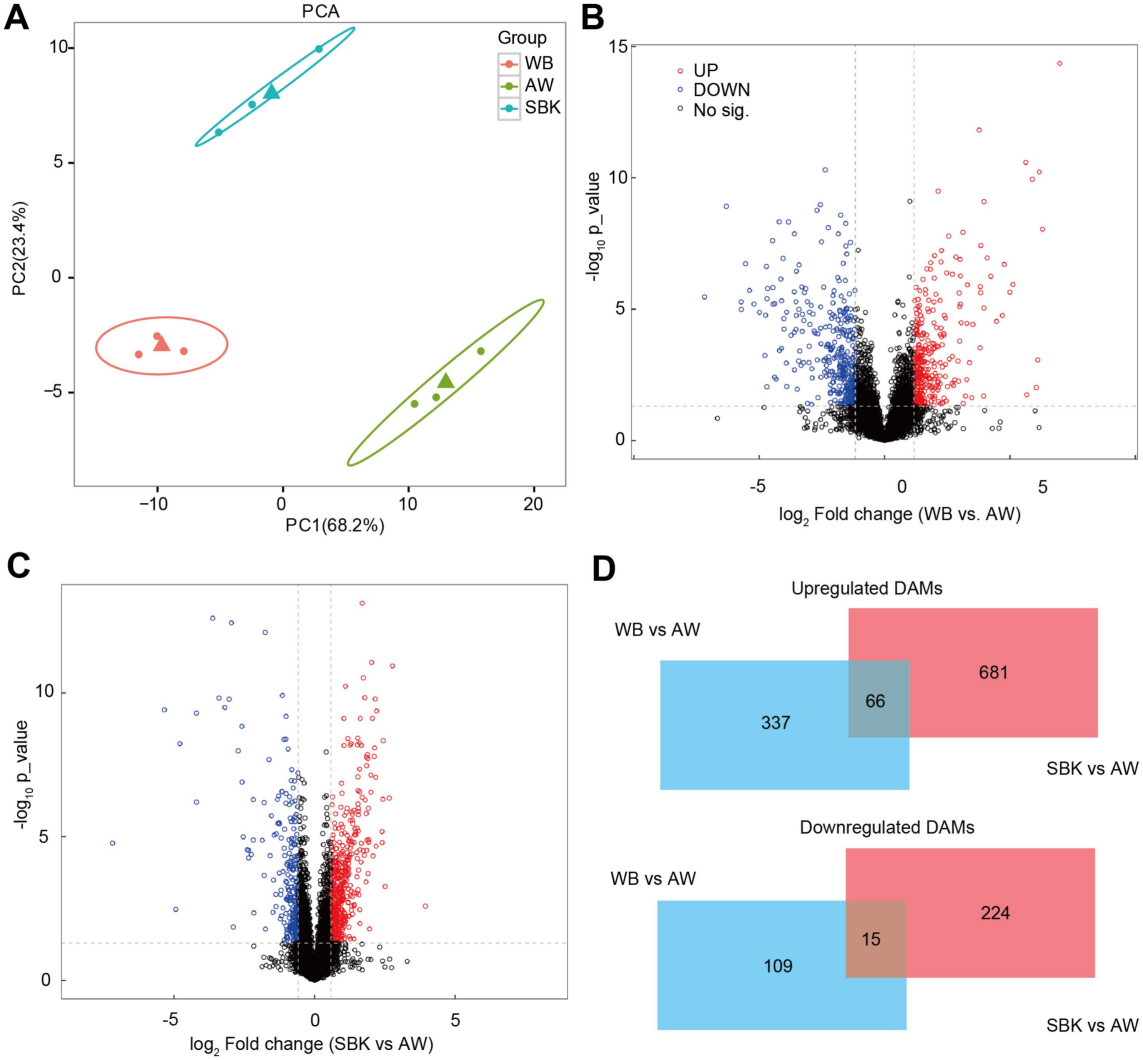
Non-targeted metabolism analysis on the mushroom fruiting bodies induced by WB or SBK relative to AW. **(A)**, principal component analysis. **(B,C)**, volcano plot representing the different abundant metabolites in the comparison of WB versus AW and SBK versus AW. **(D)**, Venn diagram represents differentially abundant metabolites in mushroom fruiting bodies induced by WB or SBK relative to AW.

**Figure 7:**
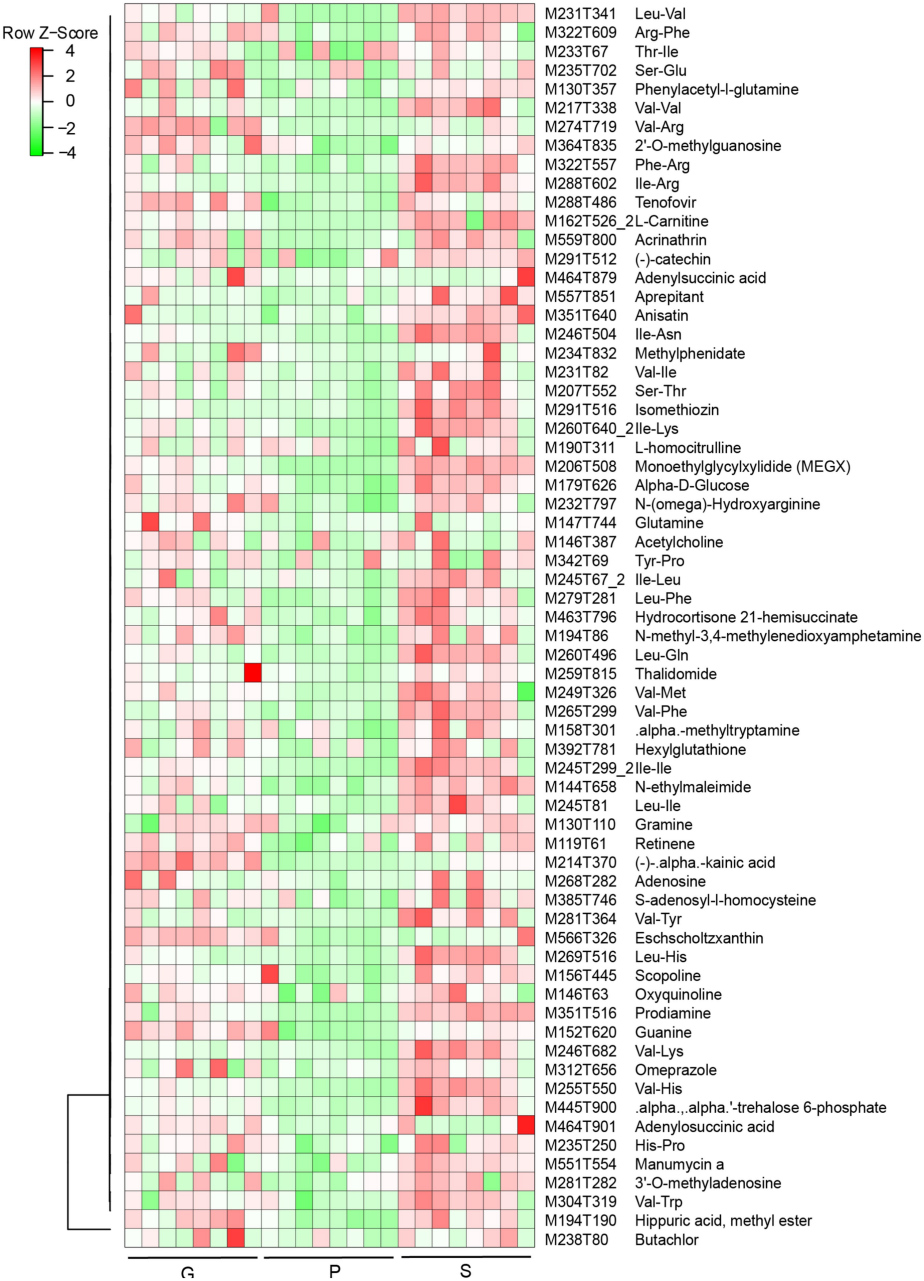
Heatmap of 66 overlapped differentially abundant metabolites (DAMs) identified in mushroom fruiting bodies induced between the two comparisons (WB vs. AW and SBK vs. AW).

Results from PCA suggested that PC1 and PC2 accounted for 68 and 23% of the variation in samples among different groups (WB, AW, and SBK) (Figure 6A). There were 403 increased DAMs and 124 decreased DAMs compared to WB vs. AW (Figure 6B). We also identified 747 increased DAMs and 239 decreased DAMs when comparing WB vs. AW and SBK vs. AW (Figures 6C,D). In addition, 66 overlapped DAMs with an increased pattern and 15 overlapped DAMs with a decreased pattern between the SBK vs. AW and WB vs. AW (Figure 6D). Furthermore, in the 66 increased DAMs that overlapped in the two comparisons, i.e., WB vs. AW and SBK vs. AW, were identified, including phenylacetyl-l-glutamine, adenylsuccinic acid, methylphenidate and N-methyl-3,4-methylenedioxyamphetamine (Figure 7).

Mushrooms are high-purine food, while humans taking high purine levels can raise uric acid levels, causing painful gout symptoms. Our studies suggest that the contents of all purines and purine derivatives determined in this study based on metabolism were similar or decreased in mushroom bodies cultivated with WB and SBK, compared to AW (Supplementary Table S5). In particular, two purine derivatives, i.e., guanine and hypoxanthine, declined at least 20% induced by WB and SBK compared to AW (Supplementary Table S5).

**Figure 8:**
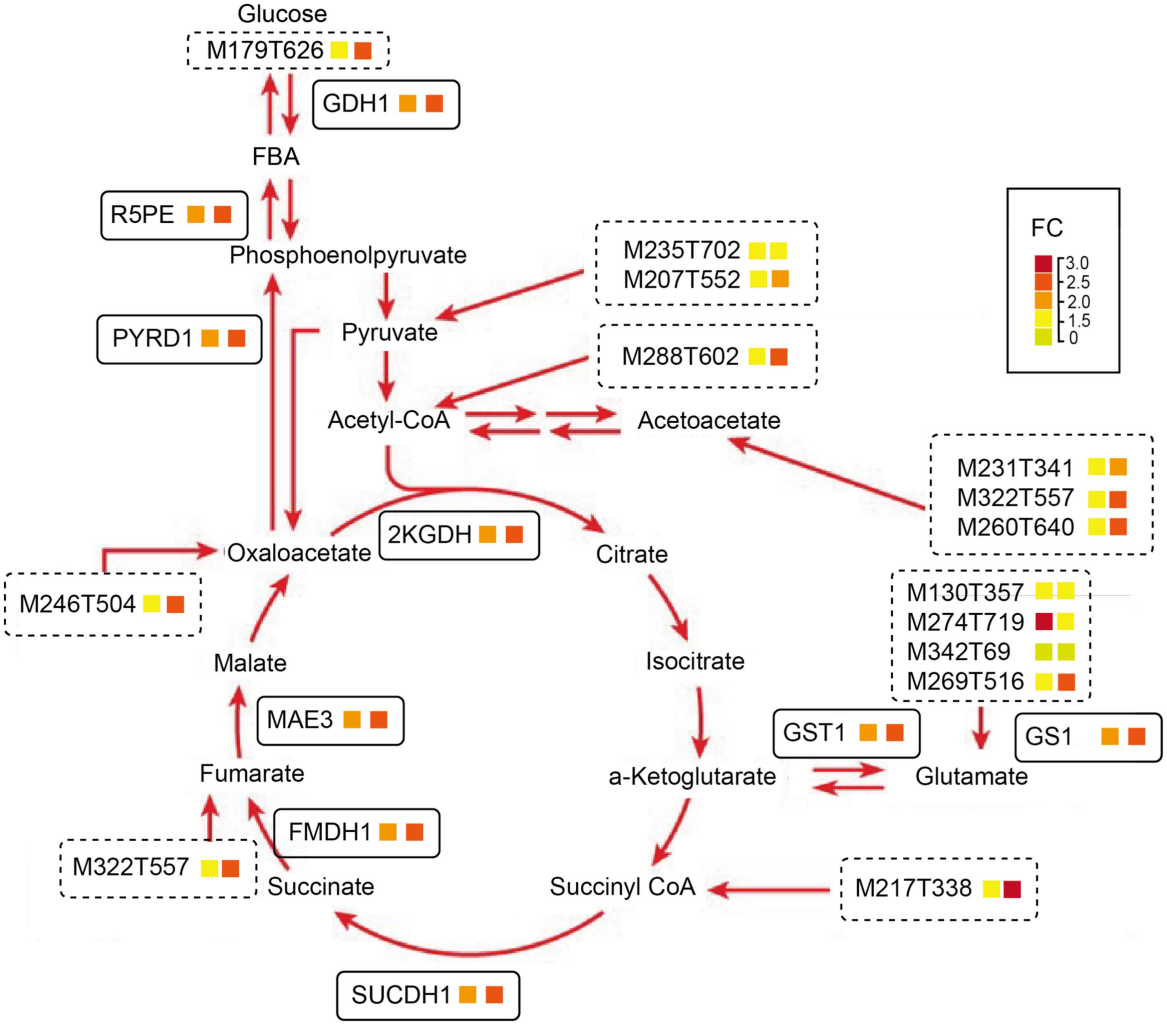
A summarized model representing changes of genes and metabolites involved in the oxidoreductase activity pathway in mushroom fruiting bodies induced by WB or SBK relative to AW is based on an integrative analysis of proteomic and metabolism. The first and second cells stand for the fold change (FC) values in WB versus AW and SBK versus AW, respectively.

In summary, based on a physiological, proteomics, and metabolism combined analysis, both WB and SBK substrates could promote the protein content relative to AW, which is related to carbohydrate assimilation metabolism, as shown by the fact that glucose (M179T626) was stimulated by both WB and SBK substrates. Following this, both gene and protein expression of GDH1, R5PE, and PYRD1 were stimulated, which facilitated the enhanced expression of genes involved in the TCA cycle, such as 2KGDH, GDH1, FMDH1, and SUCCDH1 (Figures 5C–J, 8; Supplementary Table S4). In addition, the accumulated metabolites involved in the TCA cycle coordinated with increased amino acids and derivatives, including M235T702, M207T552 (threonine), M231T341 (leucine), and M217T338 (valine) (Figures 7, 8; Supplementary Table S6). As a result, the glutamate and glutathione involved in oxidoreductase activity were dramatically enhanced, such as M130T357 (glutamine), M274T719 (arginine), M342T69 (proline), and M269T516 (histidine) (Figures 7, 8; Supplementary Table S6).

## Discussion

Cultivating edible fungi has proven to be an efficient biological method for converting agroforestry by-products, such as AW, WB, and SBK, with their abundant lignocellulosic biomass into valuable mushroom products. Many studies reported AW as a substrate ingredient for mushroom cultivation, but fewer focused on WB and SBK. In this study, we observed that these two substrates, WB and SBK, have promotive effects on nutritional profiling, including crude protein and fibers. Therefore, we combined proteomics and metabolism analysis to identify key genes and biological pathways altered by WB and SBK in mushroom fruiting bodies to uncover further the key molecular features responsible for the promotive effect. Because of two substrates, we confirmed the enhanced expression of eight genes involved in carbohydrate assimilation pathways and oxidoreductase activity. This evidence suggests that reprogrammed carbon metabolism and oxidoreductase pathways play critical roles in the enhanced response of mushrooms to both WB and SBK substrates. This study may inform targeted genetic modifications to improve the economic value of mushroom production.

### Promoted effects of WB and SBK on mushroom nutritional features

Mushrooms provide a rich array of essential nutrients such as proteins, polysaccharides, dietary fibers, vitamins, and minerals, making them an excellent dietary source (29). These nutritional features of the fruiting bodies significantly impact the commercial value and production efficiency of *L. edodes*. Different substrates can influence these attributes. Our study revealed that among the three nutritional features, only crude protein and crude fat content in mushroom fruiting bodies were significantly enhanced by WB and SBK supplementation relative to AW (Figures 1B,C). A similar enhancement in crude protein has been reported in mushroom fruiting bodies cultivated with wheat straw and brassica straw (30). The addition of apple pomace could improve crude fat content by 35% (31). These findings suggest that WB and SBK supplementation in mushroom substrates relative to AW can notably enhance total protein accumulation.

In this study, we observed a dramatic enhancement of three nutrient features, including crude protein, fatty acids, and fiber, in mushroom bodies cultivated with WB and SBK relative to AW. Therefore, our work is limited to reporting the proteomic and metabolic changes in mushroom bodies rather than substrates. There is a very complex regulation mechanism from substrates to mushroom bodies for different nutritional features, including physiological features of substrates, external environment, nutritional features conversation and transporting efficiency, etc. (32, 33). Three crude nutritional features (crude protein, fiber, and fat) show dramatically different transition efficiencies from substrates to mushroom bodies (Figures 1E–G). Constructing a causal relationship between the specific nutrients from substrates to metabolic and proteomic changes in mushroom bodies would be a very interesting topic for future research.

### Carbon metabolic pathways involved in WB and SBK stimulated effects

Carbon metabolism, the citric acid cycle (TCA cycle), and oxidoreductase metabolism are central biochemical pathways in cellular energy metabolism. Polysaccharides are initially synthesized in carbon metabolism from two types of activated glucose molecules: *UDP-glucose and ADP-glucose.* Glucose plays a central role in energy utilization. Importantly, we observed a significant increase in alpha-D-glucose (M179T626) induced by WB and SBK relative to AW in mushroom fruiting bodies (Figure 8; Supplementary Table S6). The rise in glucose directly led to high oxaloacetate (M246T504) involved in the TCA cycle through the catalysis of various enzymes, including GDH1, R5PE, and PYRD1. Particularly, the pyruvate dehydrogenase complex (PDC) acts as a multifunctional enzyme complex pivotal in aerobic respiration, bonding glycolysis to the mitochondrial oxidation of pyruvate (34). Another pivotal enzyme in carbon metabolism is Ribulose-5-phosphate-3-epimerase (R5PE). It facilitates the formation of Ru5P and plays a role in the pentose-phosphate pathway by catalyzing the reverse reaction. Intriguingly, in the microalga *Chlamydomonas reinhardtii,* R5PE activity remains unaffected by either reductive or oxidative treatments, suggesting that enzyme catalysis remains insensitive to potential redox alterations of cysteine residues (35).

### Oxidoreductase activity positively associated with WB and SBK effects

Cellular redox homeostasis is essential for maintaining many cellular processes, including oxidoreductase metabolic reactions and the response to ROS. In this regard, some amino acids, as protein components, also maintain cellular redox homeostasis (36, 37). In this study, we observed a dramatic increase in glutamine (M147T744) and hexylglutathione (M392T781) induced by both WB and SBK in mushroom fruiting bodies (Figure 8; Supplementary Table S6). Importantly, GDH1 (GAW04455.1) observed in this study is an oxidoreductase. It catalyzes glucose oxidation to hydrogen peroxide and initiates the pentose phosphate pathway. The pathway’s primary function is reducing nicotinamide adenine dinucleotide phosphate (NADP^+^) to NADPH. NADPH is then utilized to reduce oxidized glutathione (GSSG) to its reduced state (GSH), reducing mixed disulfides of glutathione and cellular proteins (38). The increased GDH1 and glutamine ensure energy production, e.g., via oxidative phosphorylation. Thus, mushrooms can maintain a relatively stable redox state (39).

### Mitochondria TCA metabolism stimulated by WB and SBK

Furthermore, the TCA cycle plays a central role in metabolism by supplying intermediates for energy production and amino acid synthesis. Mitochondria are the primary oxygen-consuming organelles in cells, housing the oxidative phosphorylation reaction, which could couple oxygen consumption by the mitochondrial respiratory chain (MRC) with the conversion of energy to the chemical form of adenosine triphosphate (ATP). In this study, we observed that many amino acids were enhanced by both WB and SBK, including M235T702 (serine) and M207T552 (threonine) derived from pyruvate, M217T338 (valine) from succinyl CoA, M274T719 (arginine), M342T69 (proline) and M269T516 (histidine) from glutamate, M322T557 (phenylalanine) and M260T640_2 (lysine) from fumarate, M288T602 (isoleucine) from Acetyl CoA, M246T504 (asparagine) from oxaloacetate (Figure 8). The accumulation of these amino acids provides an adequate ATP supply for energy metabolism, which is helpful for the complete oxidation of reactive oxygen species (40).

### No enhancements of purine derivates in mushroom bodies by WB and SBK

It is generally accepted that in mammals, purine compounds can raise uric acid levels, which then build up in the joints and cause painful gout symptoms, while mushrooms are high-purine foods (41, 42). For example, hyperuricemia (HUA) is characterized by abnormally elevated levels of serum uric acid, the product of purine metabolism (42). Our studies suggest that the contents of all purines and purine derivatives determined in this study based on metabolism were similar or decreased in mushroom bodies cultivated with WB and SBK, compared to AW (Supplementary Table S5). In particular, two purine derivatives, i.e., guanine and hypoxanthine, declined at least 20% induced by WB and SBK compared to AW (Supplementary Table S5). In addition, the yield in mushroom bodies cultivated with the two substrates was substantially increased (Figures 1H,I). This evidence indicates the importance of utilizing these two substrates to improve production and quality.

## Data Availability

The datasets presented in this study can be found in online repositories. The names of the repository/repositories and accession number(s) can be found in the article/Supplementary material.
