# Supplementary material for: Combined analysis of proteomics and metabolism reveals critical roles of oxidoreductase activity in mushrooms stimulated by wolfberry and sea buckthorn substrates
Source: Front Nutr. 2025 Mar 18;12:1543240. doi: 10.3389/fnut.2025.1543240 (PMC11958190; doi:10.3389/fnut.2025.1543240)
Supplement: Supplementary file 1 [file Data_Sheet_1.ZIP › Supplementary Figures.docx]

**Supplementary Figures**


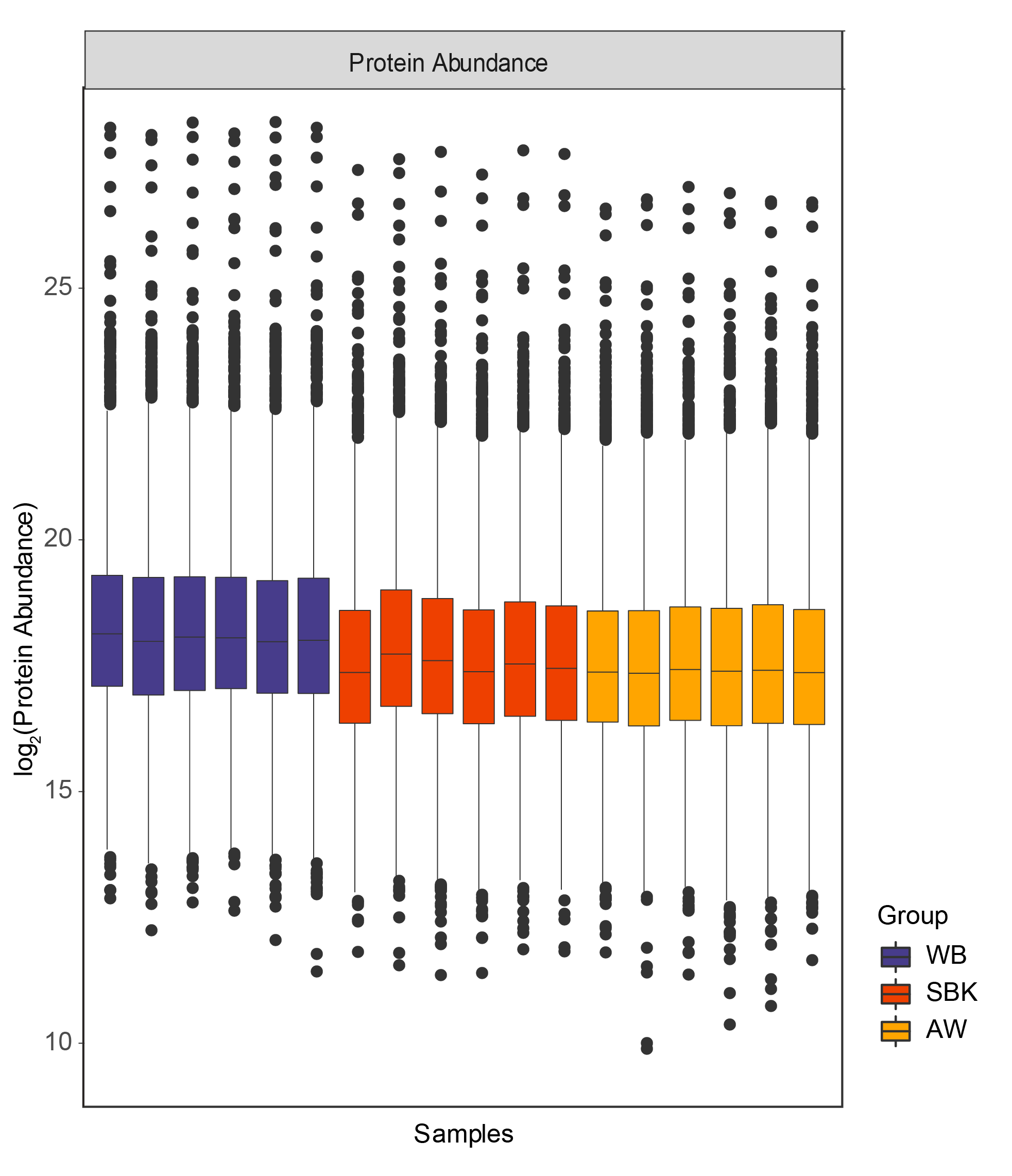


**Figure S1.** Protein abundance across different samples of mushroom fruiting bodies treated with three substrates (WB, SBK and AW).


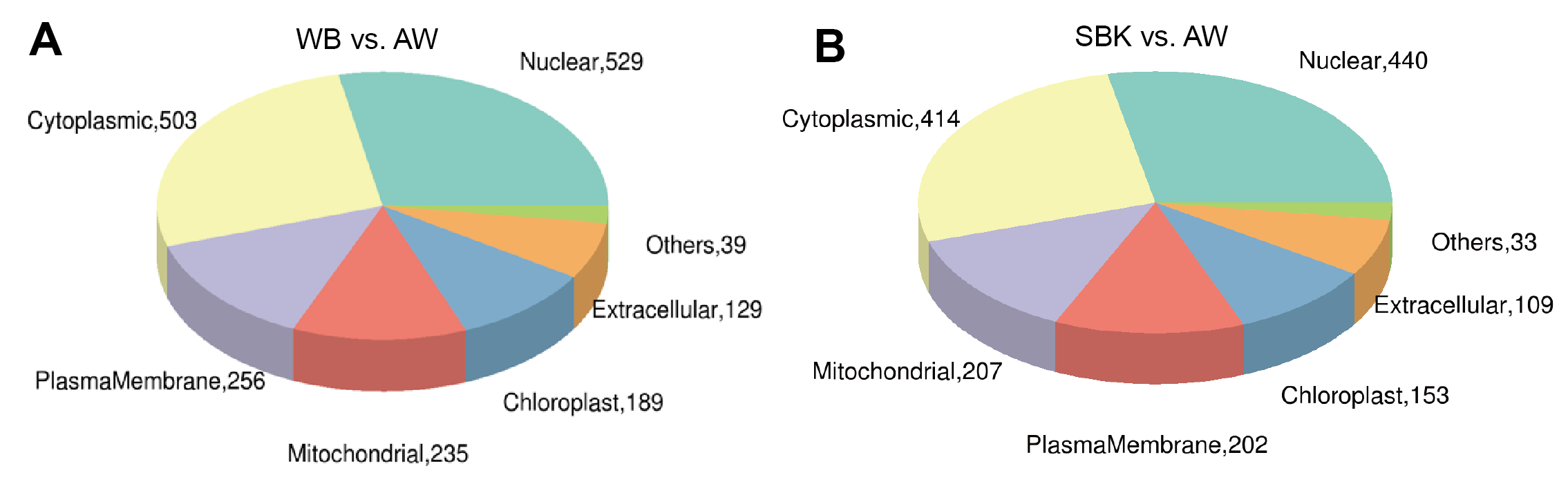


**Figure S2.** Subcellular localization of identified proteins based on proteomics. **A**, WB vs. AW. **B**, SBK vs. AW.


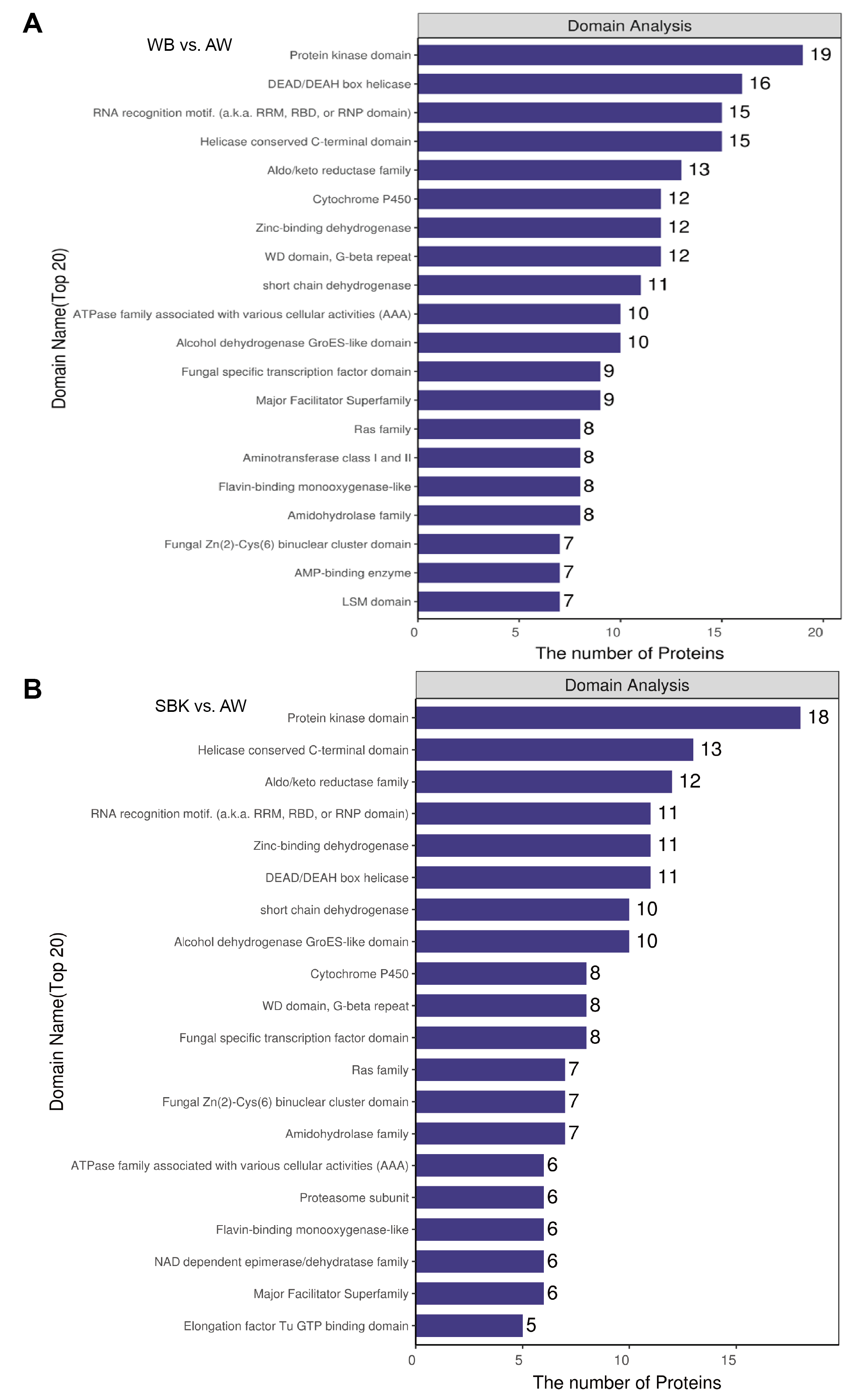


**Figure S3.** Statistical analysis on the domain enriched in the list of differentially abundant proteins induced by either WB or SBK, relative to AW. **A**, WB vs. AW. **B**, SBK vs. AW.


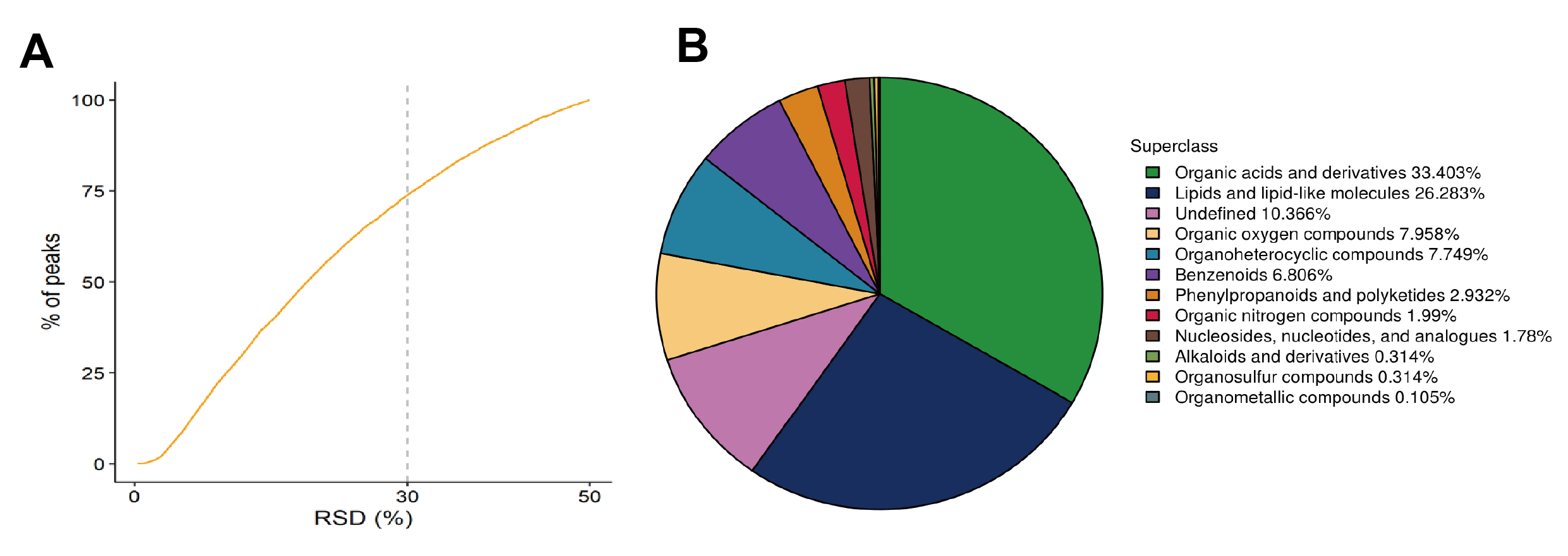


**Figure S4.** QC control and chemical taxonomy of metabolites identified across samples. **A**, relative standard deviation (RSD) curve of the ion peak abundance of the QC sample. **B**, chemical taxonomy information of metabolites identified in this study.
